# Network approach for decision making under risk—How do we choose among probabilistic options with the same expected value?

**DOI:** 10.1371/journal.pone.0196060

**Published:** 2018-04-27

**Authors:** Wei Pan, Yi-Shin Chen

**Affiliations:** 1 Institute of Physics, Academia Sinica, Taipei 115, Taiwan; 2 Department of Physics, Chung Yuan Christian University, Tao-Yuan 320, Taiwan; 3 Center for General Education, National Tsing Hua University, Hsin-Chu 300, Taiwan; 4 Tsio-Hai Waldorf School, Hsin-Chu 305, Taiwan; 5 Institute of Information Systems and Applications, National Tsing-Hua University, Hisn-Chu 300, Taiwan; 6 Department of Computer Science, National Tsing-Hua University, Hisn-Chu 300, Taiwan; Rutgers The State University of New Jersey, UNITED STATES

## Abstract

Conventional decision theory suggests that under risk, people choose option(s) by maximizing the expected utility. However, theories deal ambiguously with different options that have the same expected utility. A network approach is proposed by introducing ‘goal’ and ‘time’ factors to reduce the ambiguity in strategies for calculating the time-dependent probability of reaching a goal. As such, a mathematical foundation that explains the irrational behavior of choosing an option with a lower expected utility is revealed, which could imply that humans possess rationality in foresight.

## Introduction

Decision making under risk, *i*.*e*., all possible outcomes and the associated probabilities of the options are well known, has attracted the interests of researchers in a number of disciplines, including economics, psychology, neuroscience, and business, and has also inspired discourse on human’s rationality [1–10]. The expected utility theory established by von Neumann and Morgenstern [11] states that in a multiple choices problem, a rational decision is assumed to maximize the utility function (EU), the product of utility (*u*) and the associated probability (*p*). Many cases have shown that this criterion provides a good reference in decision making and can explain the behaviors of decision makers [3, 12–14].

However, the utility theory does not provides explanations for several behaviors in decision making. Several studies have also shown that a certain amount of people do not always take the option(s) with the maximum EU [15–21]. For instance, in experiments conducted by Kahneman and Tversky, given the choice between options with (*u*, *p*) of *A*(4000, 0.2) and *B*(3000, 0.25), 65% of the subjects took the former. However, between *C*(4000, 0.8) and *D*(3000, 1), 80% of the subjects took the latter. Here, the option (*u*, *p*) means that the subjects who take this option have a *p* chance of obtaining *u* and a (1 − *p*) chance of obtaining nothing. In other words, at least 45% of the overlapped subjects were inconsistent in responding to these two questions [22–27]. Kahneman and Tversky explained the psychological aspect of choosing *D* between *C* and *D*; in gain framing, as proposed in their prospect theory, these people were referred to as risk averse [17, 25, 28–30]. The framing effect introduced in prospect theory pointed out that people might count on different reference points in frames of gains and losses [31–33]. Moreover, people are often risk averse in decisions involving gains and are often risk seeking in decisions involving losses. These inconsistent behaviors owing to the shift of reference points in different frames are considered as irrational behaviors. Consistency is a principal aspect of the rational behaviors [34–39].

In evolution in the natural world, an act that is broadly disseminated through genes or memes should benefit, or at least not be harmful to, the actors or the offspring. In other words, an apparently detrimental action that is often observed should be beneficial in an obscure way [40]. The behavior of human beings, who are members of the ecological sphere, should also be confined by such a natural rule. Therefore, ‘inconsistent’ behaviors require a further study to find the hidden benefits and mechanisms.

Further, the applicability of the expected utility theory in decision making relies on the viability of maximizing the EU, which counts on the law of large numbers. That is, it requires a large number of people making a decision or a person making a decision a lot of times to obtain outcomes close to the expected value. However, for an individual who has aspirations other than the average, the results from many people would not be a good reference to make such a decision. The real outcome would deviate a lot from that predicted by the expected value [41–45]. Thus, new criteria other than maximizing the EU are needed.

Timing is significant as to satisfy one’s needs in time is an important issue in decision making [44–52]. With respect to time, people may have different needs in short-term or long-term. An individual may have different financial needs at different stage of life. Thus, one may need to set specific goals in the financial plans according to the ages [53]. Additionally, achieving a goal as soon as possible usually, but not always, fits the decision maker’s needs. For example, in an election campaign, the best strategy for the candidate is to reach the highest rate of support exactly on voting day in order to transform the support rate into votes−peaking too early or too late does not as effectively benefit the candidate. Thus, the reference points may change in different periods [54, 55].

In this study, we provide an alternative aspect by considering goals and the times in making decisions. It is assumed that the behaviors in decision making is meant to maximize the probability of achieving a goal during or by a designated time. As human beings have a planning nature, taking the goal and time, a concrete aim for the future, into account makes an individual’s activity meaningful [56].

This paper is organized as follows. First, a model that considers both goals and times is proposed; in this model, decisions are considered to be a process moving from the initial stage through several intermediate stages to ultimately reach the goal. These stages and the connecting paths are respectively illustrated as nodes and links in a network [57–59]. The decisions are a series of choosing a link that connects the current node to the next node until arriving to the node of goal. This process is equivalent to a walking on the constructed network, which is then further transformed into a matrix [60]. We then introduce strategies that set different weighting on choosing the available options. The outcomes of this series of decisions can be calculated by iteration from the continual multiplication of the matrices. Several strategies are raised to demonstrate how the proposed model works. Finally, we discuss the applications of the model in measuring the goal in mind and the mathematical foundation to the economic behaviors.

## Methods

In our model, the decision process is divided into three parts, (1) *Game set*, (2) *Strategy*, and (3) *Iteration*. The first part, *Game set* depends on the situation in which the decision maker is involved including the number of options and the associated (*u*, *p*). Not losing generality, we set the utility as the monetary value. According to *Game set*, one can construct a network, which resembles the diagram in Markov decision process [61–63]. The second part, *Strategy*, is new that a decision maker is freely to distribute the weights on options as to narrowly or broadly bracket choices [64]. One can construct the nodes and links of the decision network according to these two parts. We can further transform the network into a matrix and proceed the third part, *Iteration* to find the time-dependent probability of achieving the goal, **P**_**com**_(**t**). The decision maker can adjust *Strategy* to maximize **P**_**com**_(**t**) during the designated *t*. In this model, the cost is the time spent on the game.

### Game set: Construction of the network

**Fig 1:**
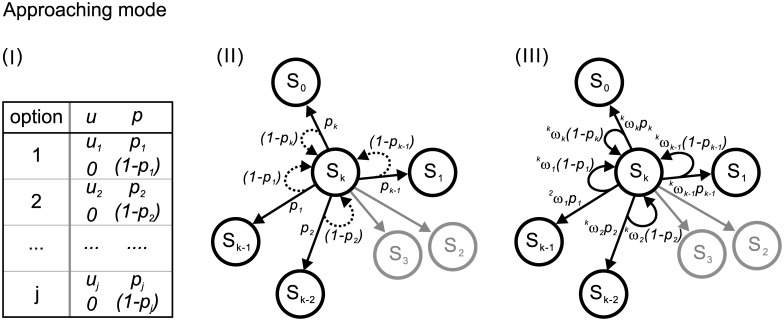
Construction of the network in a decision game of G of S_0_ in *Approaching mode*. (I) Payoff table: the decision maker who chooses option *j* has a chance of *p*_*j*_ to gain *u*_*j*_ or a chance of (1 − *p*_*j*_) to gain nothing. (II) Game set: the game set according to the payoff table in (I) is further illustrated. At S_*k*_, the decision maker has *k* options, *e*.*g*., option 1 leads to S_*k*−1_ with a probability of *p*_1_ or stay at S_0_ with a probability of (1 − *p*_*k*−1_), option 2 leads to S_*k*−2_ with a probability of *p*_2_ or stay at S_0_ with a probability of (1 − *p*_2_), …, and option *k* leads to S_0_ with a probability of *p*_*k*_ or stay at S_0_ with a probability of (1 − *p*_*k*_). (III) Weights: the decision maker at S_*k*_ can distribute the weights of ^*k*^*w*_1_, ^*k*^*w*_2_, …, and ^*k*^*w*_*k*_ on the options of (*u*_1_, *p*_1_), (*u*_2_, *p*_2_), …, and (*u*_*k*_, *p*_*k*_), respectively. Thus, the probabilities of moving from S_*k*_ to S_*k*−1_, S_*k*−2_, …, and S_0_ are ^*k*^*w*_1_ *p*_1_, ^*k*^*w*_2_ *p*_2_, …, and ^*k*^*w*_*k*_ *p*_*k*_, respectively. The one also has a probability of (^*k*^*w*_1_(1 − *p*_1_) + ^*k*^*w*_2_(1 − *p*_2_) + … + ^*k*^*w*_*k*_(1 − *p*_*k*_)) of staying at S_*k*_.

**Fig 2:**
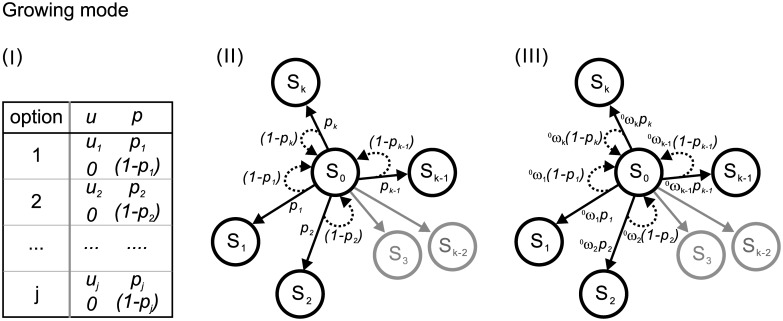
Construction of the network in a decision game of G of S_0_ in *Growing mode*. (I) Payoff table: the decision maker who chooses option *j* has a chance of *p*_*j*_ to gain *u*_*j*_ or a chance of (1 − *p*_*j*_) to gain nothing. (II) Game set: the game set according to the payoff table in (I) is further illustrated. At S_0_, the decision maker has *k* options, *e*.*g*., option 1 leads to S_1_ with a probability of *p*_1_ or stay at S_0_ with a probability of (1 − *p*_1_), option 2 leads to S_2_ with a probability of *p*_2_ or stay at S_0_ with a probability of (1 − *p*_2_), …, and option *k* leads to S_*k*_ with a probability of *p*_*k*_ or stay at S_0_ with a probability of (1 − *p*_*k*_). (III) Weights: the decision maker at S_0_ can distribute the weights of ^0^*w*_1_, ^0^*w*_1_, …, and ^0^*w*_*k*_ on the options of (*u*_1_, *p*_1_), (*u*_2_, *p*_2_), …, and (*u*_*k*_, *p*_*k*_), respectively. Thus, the probabilities of moving from S_0_ to S_0_, S_1_, S_2_, …, and S_*k*−1_ are ^0^*w*_1_ *p*_1_, ^0^*w*_2_ *p*_2_, …, and ^0^*w*_*k*_ *p*_*k*_, respectively. The one also has a probability of (^0^*w*_1_(1 − *p*_1_) + ^0^*w*_2_(1 − *p*_2_) + … + ^0^*w*_*k*_(1 − *p*_*k*_)) of staying at S_0_.

*Approaching mode* and *Growing mode* are raised for constructing the network as shown in Figs 1 and 2, respectively.

The payoff table of the decision game for the *Approaching mode* is listed in Fig 1(I). The decision maker who choose option *j* has a *p*_*j*_ chance of obtaining *u*_*j*_ that move *j* stages and a (1 − *p*_*j*_) chance of staying at the original stage. In the *Approaching mode* as shown in Fig 1(II), the stage occupied by the decision maker is denoted as S_*k*_, indicating that it requires *k* stages to reach the goal, S_0_. Thus, a series of decisions is considered a walk from S_*k*_ to S_0_ in the network. The available options correspond to the links connecting to the following nodes according to the payoff table. For a decision maker at S_*k*_, option *i* indicates an outward link pointing to S_*k*−*i*_ and a returning link associated with probabilities of *p*_*i*_ and (1 − *p*_*i*_), respectively. The value and the utility of *u*_*i*_ is set to be equal to move *i* stages in our research.

In the *Growing mode* as shown in Fig 2(II), the initial stage is denoted as S_0_. The options taken by the decision maker correspond to the links connecting to the following nodes according to the payoff table as shown in Fig 2(I). At S_0_, option *i* indicates an outward link pointing to S_*i*_ and a returning link associated with probabilities of *p*_*i*_ and (1 − *p*_*i*_), respectively. Again, the value and the utility of *u*_*i*_ is set to be equal to move *i* stages.

### Strategy matrix, W

The decision maker can distribute weights on the possible links (options). In the *Approaching mode* as shown in Fig 1(III), the decision maker can distribute a weight, ^*k*^*w*_1_ (< 1), on option *i* when she is at S_*k*_. Thus, she has a ^*k*^*w*_*i*_ *p*_*i*_ chance of moving to *S*_*k*−*i*_ and a ^*k*^*w*_*i*_(1 − *p*_*i*_) chance of staying at S_*k*_. Generally, at S_*k*_, the decision maker can set the weights of ^*k*^*w*_1_, ^*k*^*w*_2_, … ^*k*^*w*_*k*−1_, and ^*k*^*w*_*k*_ on the options 1, 2, …, *k* − 1, and *k*, respectively. Thus, the chances of moving from S_*k*_ to S_*k*−1_, S_*k*−2_, …S_1_, and S_0_ are ^*k*^*w*_1_ *p*_1_, ^*k*^*w*_2_ *p*_2_, … ^*k*^*w*_*k*−1_ *p*_*k*−1_, and ^*k*^*w*_*k*_ *p*_*k*_, respectively. The chance of staying at S_*k*_ is (^*k*^*w*_1_(1 − *p*_1_) + ^*k*^*w*_2_(1 − *p*_2_) + …^*k*^*w*_*k*−1_(1 − *p*_*k*−1_) + ^*k*^*w*_*k*_(1 − *p*_*k*_)), *i*.*e*. $\sum_{i=1}^{k}$^*k*^*w*_*i*_(1 − *p*_*i*_).

In the *Growing mod* as shown in Fig 2(III), the decision maker can distribute a weight, ^0^*w*_*i*_ (< 1), on option *i* when she is at S_0_. Thus, she has a ^0^*w*_*i*_ *p*_*i*_ chance of moving from S_0_ to *S*_*i*_ and a ^0^*w*_*i*_(1 − *p*_*i*_) chance of staying at S_0_. Generally, at S_0_, the decision maker can set the weights of ^0^*w*_1_, ^0^*w*_2_, … ^0^*w*_*k*−1_, and ^0^*w*_*k*_ on the options 1, 2, …, *k*−1, and *k*, respectively. Such that, the chances of moving from S_0_ to S_1_, S_2_, …S_*k*−1_, and S_*k*_ are ^0^*w*_1_ *p*_1_, ^0^*w*_2_ *p*_2_, … ^0^*w*_*k*−1_ *p*_*k*−1_, and ^0^*w*_*k*_ *p*_*k*_, respectively. The chance of staying at S_0_ is (^0^*w*_1_(1 − *p*_1_) + ^0^*w*_2_(1 − *p*_2_)+ … ^0^*w*_*k*−1_(1 − *p*_*k*−1_) + ^0^*w*_*k*_(1 − *p*_*k*_)), *i*.*e*. $\sum_{i=1}^{k}$^0^*w*_*i*_(1 − *p*_*i*_).

Since the outward links are conservative, the total of the weights is set as 1, *i*.*e*., $\sum_{i=1}^{j}$^*j*^*w*_*i*_ = 1 for any *j* in both modes. A strategy in a decision corresponds to how the decision maker distribute the weights, ^*j*^*w*_*i*_, on the available links at S_*j*_. For simplicity, we take *Approaching mode* in the following sections. A decision maker at S_*i*_ distributes the weight on the link, (*u*_*m*_, *p*_*m*_) and is denoted as ^*i*^*w*_*m*_, which indicates moving *m* stages to S_*i*−*m*_ with a probability of *p*_*m*_ or staying at S_*i*_ with a probability of (1 − *p*_*m*_). A strategy matrix **W** is set when all elements, *b*_*ij*_ are set, which are equivalent to ^*i*^*w*_*i*−*j*_.

### Matrices

The network of a decision game can be transformed into a game matrix **Q** [60]. The **Q** is a (*k* + 1) × (*k* + 1) matrix with the elements, *q*_*ij*_ are the probability connecting S_*i*_ to S_*j*_. Additionally, the distribution of the weights on each option can be represented in a strategy matrix **W**. In our game setting, **Q** is determined by the decision game and **W** depends on the decision maker’s strategy. They are mutually independent. The **Q** and the **W** can be further composed into a matrix **A**. The correlation of these matrices are shown in Eq 1, in which “∘” denotes the operation of the Hadamard product, *a*_*ij*_ = *q*_*ij*_ ⋅ *b*_*ij*_ for *i* > *j*. The diagonal elements of **A** are set in **D**, which contains only diagonal elements, *i*.*e*., *d*_*ii*_ = *a*_*ii*_ and *d*_*ij*_ = 0 *for i* ≠ *j*.

$$\begin{array}{c} A=Q\;\circ \;W+D \end{array}$$ (1)

Walking on the network can be further transformed into an iteration of a transition matrix, **A**. The element *a*_*ij*_ indicates the transition probability from S_*i*_ to S_*j*_ [60]. Thus, **A** is a (*k* + 1) × (*k* + 1) matrix with elements indexing from *a*_00_ to *a*_*kk*_. Those *a*_*ij*_ are i ^*i*^*w*_(*i*−*j*)_ *p*_(*i*−*j*)_ for *i* > *j*. The diagonal elements, *a*_*ii*_ corresponding to the self-linking links, are $\sum_{m=1}^{i}$^*i*^*w*_*m*_(1 − *p*_*m*_). The left part of **A** and the associated elements *a*_*ij*_ are shown in Eqs 2 and 3, respectively.

$$A=\left[\begin{array}{ccccc} 1 & 0 & 0 & 0 & \ldots \\ {}^{1}w_{1}p_{1} & {}^{1}w_{1}(1-p_{1}) & 0 & 0 & \ldots \\ {}^{2}w_{2}p_{2} & {}^{2}w_{1}p_{1} & \begin{array}{c} {}^{2}w_{2}(1-p_{2})+\\ {}^{2}w_{1}(1-p_{1}) \end{array} & 0 & \ldots \\ {}^{3}w_{3}p_{3} & {}^{3}w_{2}p_{2} & {}^{3}w_{1}p_{1} & \begin{array}{c} {}^{3}w_{3}(1-p_{3})+\\ {}^{3}w_{2}(1-p_{2})+\\ {}^{3}w_{1}(1-p_{1}) \end{array} & \ldots \\ \ldots & \ldots & \ldots & \ldots & \ldots \end{array}\right]$$ (2)

$$a_{ij}=\{\begin{array}{cc} {}^{i}w_{\left(i-j\right)}p_{\left(i-j\right)} & for\;i>j\\ \Sigma_{m=1}^{i}{\;}^{i}w_{m}\left(1-p_{m}\right) & for\;i=j \end{array}$$ (3)

$$\begin{array}{ll} \sum_{m=1}^{i}a_{im} & {=}^{i}w_{m}p_{m}{+}^{i}w_{m-1}p_{m-1}{+}^{i}w_{m-2}p_{m-2}+\ldots {+}^{i}w_{1}p_{1}\\ & {+}^{i}w_{m}\left(1-p_{m}\right){+}^{i}w_{m-1}\left(1-p_{m-1}\right){+}^{i}w_{m-2}\left(1-p_{m-2}\right)+\ldots .{+}^{i}w_{1}\left(1-p_{1}\right)\\ & {=}^{i}w_{i}{+}^{i}w_{i-1}{+}^{i}w_{i-2}+\ldots .\\ & =\sum_{m=1}^{i}{\;}^{i}w_{m}\\ & =1 \end{array}$$ (4)

The sum of each row of **A** is 1 as shown in Eq 4, indicating that **A** is not only a lower triangular but also a stochastic matrix.

### Iteration: State vector and iteration relation

We then introduce a state matrix, **R**(*t*), [*r*_0_, *r*_1_, *r*_2_, …, *r*_*k*_] with dimension of 1 × (*k* + 1). The elements *r*_*i*_(*t*) indicate the probability of a decision maker staying at S_*i*_ in *t*. Therefore, a decision maker begins at S_*k*_, which corresponds to **R**(0) of [0, 0, 0, …, 1]. The first element, *r*_0_(*t*), is called **P**_**com**_(**t**), the probability that a decision maker arrives at S_0_ within *t*. The next state, *r*_0_(*t* + 1), is generated from the product of *r*_0_(*t*) and *a*_*ij*_ as shown in Eq 5. Thus, the state matrix **R**(*t*) fits the iteration relation as shown in Eq 6. The decision maker eventually arrives at S_0_, which corresponds to **R**(∞), [1, 0, 0, …, 0].

$$\begin{array}{c} \begin{array}{cc} r_{i}\left(t+1\right) & =r_{0}\left(t\right)a_{0i}+r_{1}\left(t\right)a_{1i}+r_{2}\left(t\right)a_{2i}+\cdots +r_{k}\left(t\right)a_{ki}\\ & =\sum_{m=0}^{k}r_{m}\left(t\right)a_{mi} \end{array} \end{array}$$ (5)

$$\begin{array}{c} \begin{array}{cc} R(t+1) & =R(t)A\\ & =R\left(t-1\right)A^{2}\\ & =R\left(t-2\right)A^{3}\\ & =\ldots \\ & =R\left(0\right)A^{t+1} \end{array} \end{array}$$ (6)

We then examine the solvability to find the best strategy to satisfy the decision maker. In a decision game beginning at S_*k*_, the number of unknown variables in **W** is *k*(*k* + 1)/2. There are (*k* + 1) constraints including the sum of each row of **W** and the desired $r_{0}^{c}$. There are still *k*(*k* + 1)/2 − (*k* + 1) undetermined variables. The problem can be analytically solved when *k* is less than 2. For *k* > 2, one can construct a trail **W** and then obtain the *r*_0_(*t*) resulting from the iteration relation in Eq 6. The decision maker may try several **W**s in order to determine strategies that satisfy the needs.

### Strategy comparison

At S_*k*_, the decision maker has *k* number of options from (*u*_1_, *p*_1_), (*u*_2_, *p*_2_), (*u*_3_, *p*_3_), …, to (*u*_(*k*−1)_, *p*_(*k*−1)_), and (*u*_*k*_, *p*_*k*_) linking to S_(*k*−1)_, S_(*k*−2)_, S_(*k*−3)_, …, to S_1_, and S_0_, respectively. For resolving the ambiguity raised in Abstract, the expected utility for each option is set as 1, *i*.*e*., (*u*_*i*_, *p*_*i*_) is set as $\left(i,\frac{1}{i}\right)$ linking from S_*k*_ to S_*k*−*i*_. This setting is also for comparison of different strategies choices among options without privilege. In this paper, we raise strategy examples: *fix*, *min k*, *max k*, *risky*, *random*, and *safe*.

**Fig 3:**
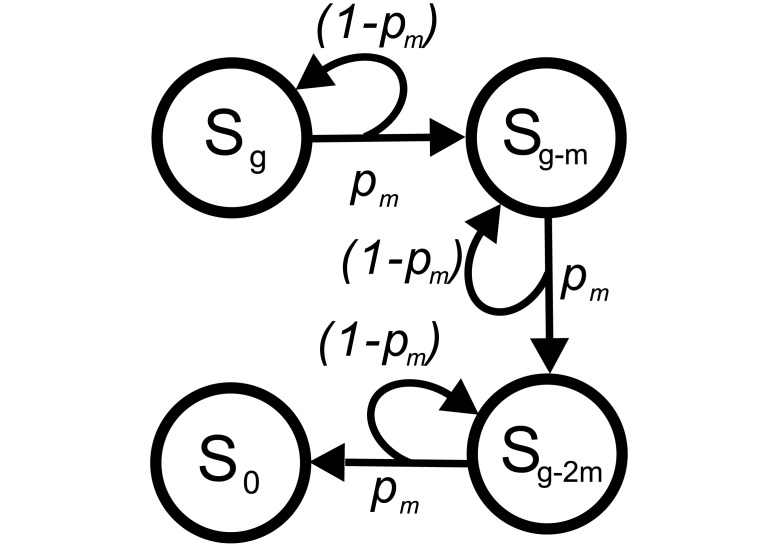
The network of the *fix* strategy choosing (*m*, *p*_*m*_). For simplicity, the **G** is set as 3*m* to demonstrate the network. The decision maker only passes the stages with indexes of (*g* − *nm*), where *m* = *g*/*u* and *n* = 0, 1, 2, *or* 3, *i*.*e*., 3 stages from S_*g*_ to S_0_. That is, there are only four stages with this strategy: S_*g*_, S_*g*−*m*_, S_*g*−2*m*_, and S_0_ (or S_3*m*_, S_2*m*_, S_*m*_, and *S*_0_, respectively).

The *fix* strategy indicates that a decision maker always chooses a certain option until the end of the game as shown in Fig 3. Assuming that the decision maker takes the option of (*m*, *p*_*m*_), only *g*/*m* stages are needed to arrive at S_0_.

**Fig 4:**
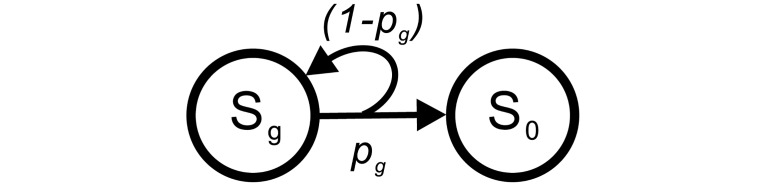
The network of the *max k* strategy in a decision game of G of *g*. The decision maker always chooses the option with the highest utility, *i*.*e*., (*g*, 1/*g*), a special case of the *fix* strategy. Thus, she either stays at S_*g*_ or moves to S_0_. The intermediate stages, S_*g*−1_ to S_1_, are not reachable.

A decision maker taking the *min k* strategy always chooses the safest option, *i*.*e*., (1, 1). In other words, the decision maker moves one stage each time. In a game with a goal (**G**) of *g*, it takes *g* stages to reach **G**. This provides us a baseline for comparison with other strategies. With the *max k* strategy, the decision maker always chooses the riskiest option, *i*.*e*., the one with the highest utility, (*g*, 1/*g*), as shown in Fig 4. The decision maker either stays at the very beginning, S_*g*_, or moves to S_0_ with a probability of (1 − 1/*g*) or 1/*g*, respectively. Both *min k* and *max k* are special cases of the *fix* strategy. With the *risky* strategy, the decision maker places more weight on the high utility options. In other words, at any stage during the game, S_*k*_, she chooses the option of (*k*, 1/*k*) in a probabilistic manner, which is in proportion to the utility, *k*. With the *safe* strategy, the decision maker places more weight on the options with high probability. That is, she chooses the option of (*k*, 1/*k*) at S_*k*_ in a probabilistic manner, which is in proportion to the probability, 1/*k*. With the *random* strategy, the decision maker chooses the option in a random manner in all stages. That is, she weights all option equally. The **W**s for the strategies, *risky*, *random*, and *safe* are listed in Table 1. Note that the real time can be estimated by multiplying *t* with an approximated period *τ* to make a decision, *i*.*e*., *tτ*.

**Table 1:** Strategies in a decision game at S_*i*_. Pr: (k, 1/k) is the probability to choose (k, 1/k).

Strategy	Pr: (k, 1/k)	^*i*^*w*_*k*_	*a*_*ij*_, *i* > *j*
*risky*	∝ *k*	2*k*/*i*(*i* + 1)	*a*_*i*, *j*_ = 2/*i*(*i* + 1)
*random*	1/*i*	1/*i*	1/*i*(*i* − *j*)
*safe*	∝ 1/*k*	$1/(k\sum_{m=1}^{i}\frac{1}{m})$	$1/({\left(i-j\right)}^{2}\sum_{m=1}^{i}\frac{1}{m})$

The results are calculated by the iteration relation, as shown in Eq 6, to see how *r*_0_(*t*) evolves with the associated **W**. It can also be performed by computer simulation. Because of the law of large numbers, both methods should produce consistent outcomes. Therefore, we only show the results from the calculations.

## Results

The results are presented both in the time-dependent cumulative distribution function (CDF, **P**_**com**_(**t**)) of the probability of achieving the goals and the associated time-dependent probability mass function (PMF, **P**(**t**)).

### Fixed option strategy

We first show the outcomes of the *fix* strategy. In a decision game with **G** of *g*, the node number is reduced from (*g* + 1) to $\left(\frac{g}{m}+1\right)$ when the decision maker takes (*m*, *p*_*m*_) only, as shown in Fig 3. The dimension of the associated matrix **A** is also reduced from (*g* + 1) × (*g* + 1) to $\left(\frac{g}{m}+1\right)\times \left(\frac{g}{m}+1\right)$. Thus, there are only two outward links for each stage: the one linking to the next stage and the other linking to the original stage. The associated **A** becomes a *band matrix* in which the non-zero elements locate only in the main diagonal and the first diagonal below, as shown in Eq 7.

$$\begin{array}{c} \left[\begin{array}{ccccc} 1 & 0 & 0 & 0 & \ldots \\ p & \left(1-p\right) & 0 & 0 & \ldots \\ 0 & p & \left(1-p\right) & 0 & \ldots \\ 0 & 0 & p & \left(1-p\right) & \ldots \\ \ldots & \ldots & \ldots & \ldots & \ldots \end{array}\right] \end{array}$$ (7)

**Fig 5:**
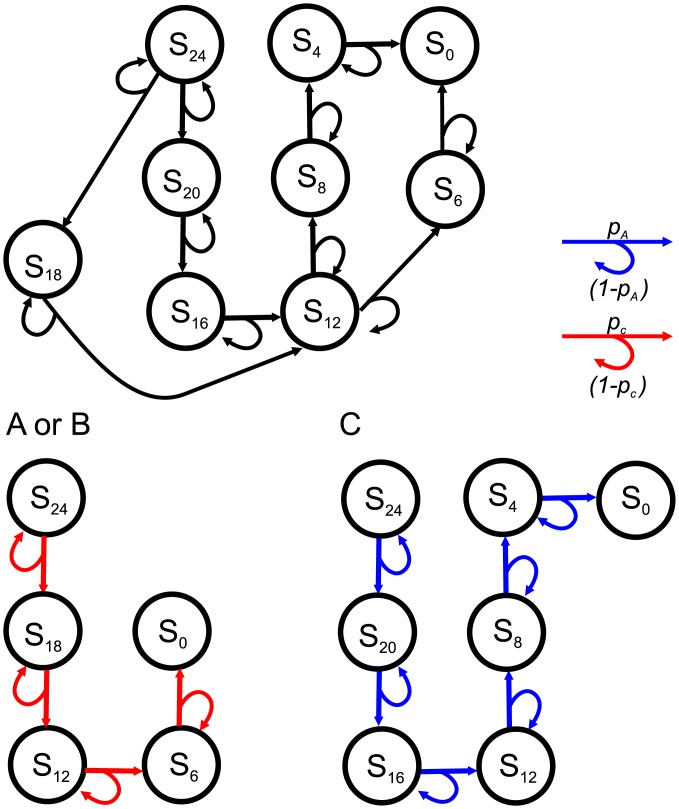
The network of the a decision game of G of 24 with options of $A(6,\frac{1}{3})$ (or *B*(6, 0.3)) and *C*(4, 0.5). The network can be decoupled when a decision maker takes the *fix* strategy. For a decision maker that chooses *A* (or *B*), the network becomes a four-stage network linking from S_24_ to S_0_ through S_18_, S_12_, and S_6_. For a decision maker that chooses *C*, the network becomes a six-stage network linking from S_24_ to S_0_ through S_20_, S_16_, S_12_, S_8_, and S_4_.

The networks of the *fix* strategies with *u* of 4 and 6 in a decision game of **G** of 24 are shown in Fig 5A and 5B, respectively. For the decision maker that chooses *C*(4, 0.5), the network becomes a six-stage link from S_24_ to S_0_ through S_4*n*_, where *n* ∈ *integers*. For the decision maker that chooses *B*(6, 0.3), the network becomes a four-stage link from S_24_ to S_0_ through S_6*n*_, where *n* ∈ *integers*. From the aspect of networks, the decision involves comparing a six-stage route with higher probability of moving to a four-stage route with lower probability of moving.

**Fig 6:**
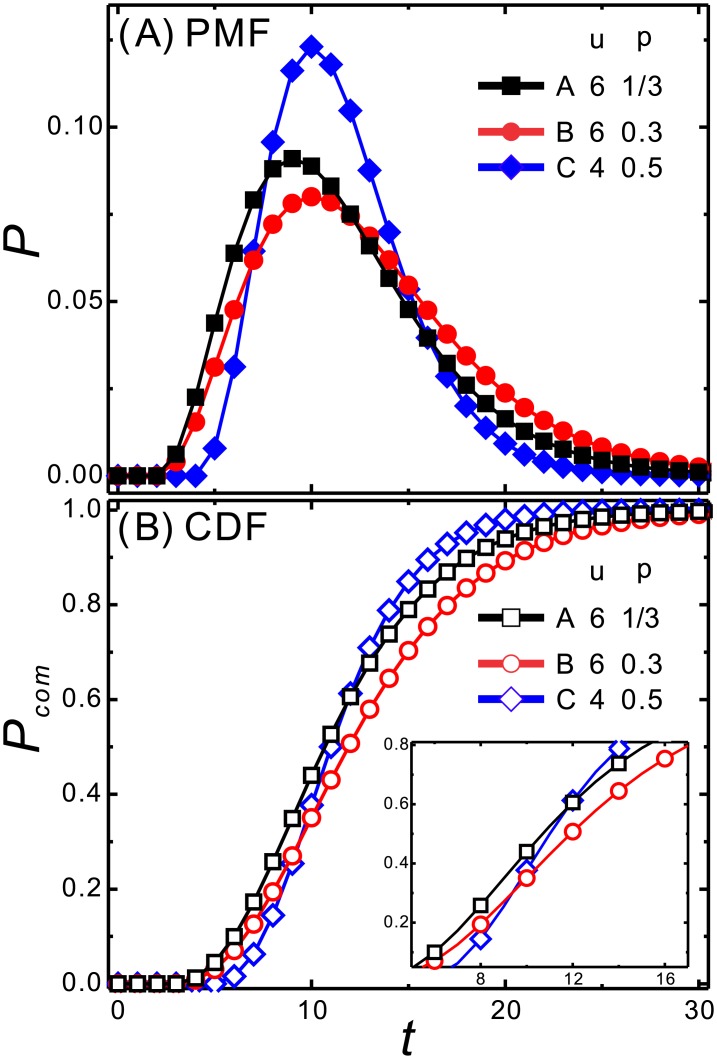
Comparison of the *fix* strategies with different expected utilities in a decision game of G of 24. The options are $A(6,\frac{1}{3})$, *B*(6, 0.3), and *C*(4, 0.5), denoted by the black square, red circle, and blue diamond, respectively. The PMF and CDF curves are shown in (A) and (B), respectively. Between *A* and *B*, where Δ*u*Δ*p* > 0, it is found that choosing *A* is dominant because **P**_**comA**_ > **P**_**comB**_ in the whole *t* range shown in (B). Between *A* and *C*, which have the same expected utility, the two curves interlace at *t*_*int*_ near 12. For *t* < *t*_*int*_, *A* is preferred, whereas for *t* > *t*_*int*_, *B* is preferred. Between *B* and *C*, which have different expected utilities, the two curves interlace at *t*_*int*_ near 10. For *t* < *t*_*int*_, *B* is preferred, whereas for *t* > *t*_*int*_, *C* is preferred. Therefore, between the options in which Δ*u*Δ*p* < 0, the preferred option becomes *t* dependent.

The game shown in Fig 5 is taken as an example for comparisons of (1) options with the same expected utility, $A(6,\frac{1}{3})$ and *C*(4, 0.5), and, (2) options with different expected utilities, *B*(6, 0.3) and *C*(4, 0.5). In Fig 6B, the CDF curves of options of *A* and *C* interlace at *t*_*int*_ of 12. This indicates that on average, both options take 12 stages to achieve the goal because the expected utility for both is 2. In the region of *t* < *t*_*int*_, **P**_**comA**_ is larger than **P**_**comC**_, which indicates that *A* is more preferable than *C* in this region. The situation is reversed in the region of *t* > *t*_*int*_, where **P**_**comC**_ is larger than **P**_**comA**_, indicating that option *C* is more preferable than *A*. The result demonstrates that options with the same expected utilities lead to different probabilities of achieving the goal. The riskier option *A* leads to a higher probability of achieving the goal in the earlier period, whereas the safer option *C* leads to a higher probability of achieving the goal in the later period. This is also found in the PMF curves that the peak positions are in the order of *A* < *C* in Fig 6A.

By introducing the goal, the ambiguity of choosing the options with the same expected utilities is eliminated. In other words, a decision maker willing to achieve an urgent goal can take the option with a larger *u*, *i*.*e*., a risky option; a decision maker willing to achieve a goal in the future can take the option with larger *p*, *i*.*e*., a safe option.

We compare the results of choosing the options with different expected utilities, *B*(6, 0.3) and *C*(4, 0.5) in Fig 6. The two CDF curves of *B* and *C* interlace at *t*_*int*_ near 10 in (B). For *t* < *t*_*int*_, that **P**_**comB**_ is larger than **P**_**comC**_ indicates *B* is preferred in the earlier region. For *t* > *t*_*int*_, that **P**_**comC**_ is larger than **P**_**comB**_ indicates *C* is preferred in the later region. Again, taking option *C*, which has a higher expected utility, is preferable for a decision maker willing to take a longer time to achieve her goal. However, for a decision maker wanting to achieve her goal in a shorter period, taking *B* is preferable, even though the corresponding expected utility is low. This might provide a mathematical explanation for the behavior of choosing the option with a low expected utility, which can be interpreted as the decision maker wishing to satisfy her desire quickly.

Comparing the CDF curves of *A* and *B* in (B), it is found that **P**_**comA**_ is larger than **P**_**comB**_ in the whole *t* range investigated. The ambiguity in decision making comes from when Δ*u*Δ*p* < 0 ((*u*_*i*_ − *u*_*j*_)(*p*_*i*_ − *p*_*j*_) < 0). This also results an interlace in the CDF curves. In other words, there exists a dominant option when *p*_*i*_ ⩾ *p*_*j*_ *or u*_*i*_ ⩾ *u*_*j*_ and Δ*u*Δ*p* > 0. The criterion of the preferable option using the *fix* strategy can be formulated as Eq 8.

$$\begin{array}{c} \left(u_{X}⩾u_{Y}\;\vee \;p_{X}⩾p_{Y}\right)\;\wedge \;e_{X}>e_{Y}\;\;⟺\;\;X≽Y \end{array}$$ (8)

### Baseline for comparison: The *min k* strategy

**Fig 7:**
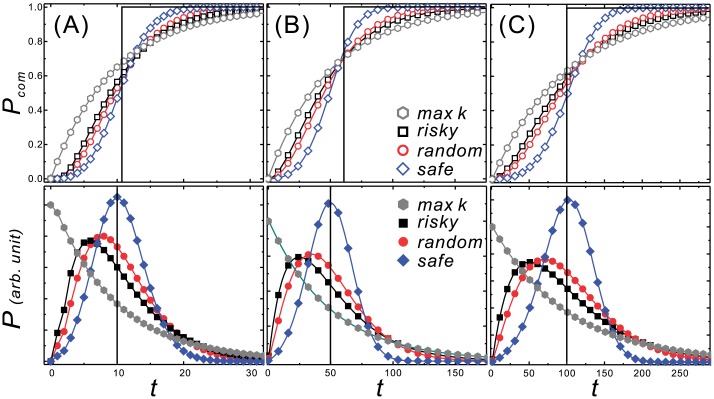
The CDF (P_com_, upper panel) and PMF (P, lower panel) curves for the strategies of *max k* (grey hexagon), *risky* (black square), *random* (red circle), *safe* (blue diamond), and *min k* (black line) to reach the goals of (A) 10, (B) 50, and (C) 100. The **P**_**com**_ curves interlace near *t*_*int*_ of 12, 60, and 120 for (A), (B), and (C), respectively. In the regime of *t* < *t*_*int*_, the **P**_**com**_ are in the order of *max k*, *risky*, *random*, *safe*, and *min k*, indicating that the *max k* strategy leads to the highest probability of achieving a goal as soon as possible. However, in the regime of *t* > *t*_*int*_, the **P**_**com**_ are in reverse order, indicating that the *min k* strategy leads to the highest probability of achieving the goal at a later time. The averages of all these strategies in (A), (B), and (C) are 10, 50, and 100, respectively.

A decision maker who takes the *min k* strategy always chooses the option of (1, 1) despite the stage at which she steps in. It takes *g* stages to move from S_*g*_ to S_0_ since this option consistently leads the decision maker to advance one stage each time. The **P** as a function of *t* for this strategy is a delta function with non-zero value at *g* and the associated **P**_**com**_ is a step function with a value of 1 for *t* ⩾ *g*, as shown in the black lines in Fig 7A, 7B and 7C. This indicates that the decision maker will not reach the goal until *t* = *g*. This can serve as a baseline for comparison with the other strategies.

### The riskiest decision maker: The *max k* strategy

The *max k* strategy can be considered a special case of the *fix* strategy where the riskiest option is always taken. The network for the *max k* strategy in a game with **G** of *g* is illustrated in Fig 4. The associated matrix is shown in Eq 9. The state **R** is a 1 × 2 row vector with an initial state **R**(0) of [0, 1], as shown in Eq 10. The **P**_**com**_ can be deduced to a function of *t*, as shown in Eq 11. The outcome of this strategy is shown in the following section.

$$\begin{array}{c} \left[\begin{array}{cc} 1 & 0\\ 1/g & \left(1-1/g\right) \end{array}\right] \end{array}$$ (9)

$$\begin{array}{c} R\left(t\right)=\left[\begin{array}{cc} 0 & 1 \end{array}\right]{\left[\begin{array}{cc} 1 & 0\\ 1/g & \left(1-1/g\right) \end{array}\right]}^{t} \end{array}$$ (10)

$$\begin{array}{c} \begin{array}{cc} & P_{com}\left(t\right)\\ & =r_{0}\left(t\right)\\ & =\frac{1}{g}\left[1+\left(1-\frac{1}{g}\right)-{\left(1-\frac{1}{g}\right)}^{2}+{\left(1-\frac{1}{g}\right)}^{3}+\ldots +{\left(1-\frac{1}{g}\right)}^{t-1}\right]\\ & =\frac{1}{g}\frac{1-{\left(1-1/g\right)}^{t}}{1-(1-1/g)}\\ & =1-{\left(1-1/g\right)}^{t} \end{array} \end{array}$$ (11)

### Comparison of the strategies

The CDF and the PMF as functions of *t* for the exemplified strategies in decision games with **G** of 10, 50, and 100 are shown in Fig 7A, 7B and 7C, respectively.

The outcomes exhibit scalability in that the positions of the peaks of the curves for the strategies investigated are in proportion to the **G**. For example, the peaks of the curves for the *safe* strategy are at 10, 50, and 100 for the **G** of 10, 50, and 100, respectively. Therefore, we will only describe the results from Fig 7A in details as the other two results for **G** of 50 and 100 exhibit the same qualitative properties.

The CDF curves are shown in the upper panels. The curves for *max k*, *risky*, *random*, and *safe* interlace at *t*_*int*_ near 12, 60, and 120 for **G** of 10, 50, and 100, respectively, which also show scalability. In the range of *t* < *t*_*int*_, the **P**_**com**_ are in the order of *max k* > *risky* > *random* > *safe* > *min k*. This result indicates that the riskier strategies lead to a higher probability of achieving the goal by *t*_*int*_. However, in the range of *t* > *t*_*int*_, the order is reversed. This indicates that the safer strategies have a higher probability of achieving the goal in the long run.

The PMF curves for the exemplified strategies are very different although the expected utility for the options are all the same. The peak position indicates that the majority of decision makers who take that strategy achieve their goal during that period. Among these strategies, the peak positions are in the order of *max k* < *risky* < *random* < *safe* ≃ *min k* as long as the *t* increases. This provides suggestions for decision makers who wish to achieve a goal during a designated period. With a **G** of 10, for example, for decision makers who wish to achieve their goals during 8 < *t* < 12, the *safe* strategy is recommended, whereas for those wish to achieve their goal during 4 < *t* < 10, the *risky* strategy is recommended.

In our game set, the expected utilities for all options are the same, as are the resulting average times to reach the goal. This is also plausible according to the law of large numbers. Thus, in the CDF curves shown in Fig 7, the strategy that leads decision makers to a higher probability of achieving a goal in an early period also leads them to a lower probability of achieving the goal in a late period. Therefore, a decision maker can set strategies, *i*.*e*., construct the **W** and apply our method to find satisfactory strategies according to the resulting **P**_**com**_(**t**) and **P**(**t**).

## Discussions

### Expected utility revisited

No matter which strategy a decision maker takes, the average *t* to reach the goal is the same, which is also guaranteed by the law of large numbers. However, the resulting distribution of **P**_**com**_(**t**) is highly dependent on the various strategies. By taking the goal and time into account, our results show that the expected utility is not the sole criterion, even though it is often used in evaluating strategies in decision making under risk. For a decision maker who wants to achieve a goal as soon as possible, riskier options are preferable than safer options, even though the former may have a lower expected utility. Therefore, the criteria for a rational decision need to be reconsidered.

Rationality often implies that a decision maker should be consistent. In other words, a decision maker who takes the option with the maximum expected utility in this question, should also take the one with the maximum expected utility in another question. According to our results, this inconsistency might imply that people do not maximizing the expected utility, but the **P**_**com**_(**t**). That is, in some circumstances, a decision maker may wish to accomplish a goal earlier, and in other circumstances, she may wish to accomplish a goal later. Furthermore, goals might vary under different circumstances. Inconsistency in answering the decision question might not exhibit the irrationality, but rather reveal a different goal-time consideration. Therefore, the meaning of rational choice needs to be redefined.

### Measurement of goal in mind

In our model, a decision maker should consider both the goal and time when choosing a strategy, as well as the distribution of the weights on options. As in the results shown in Fig 6, a decision maker who chooses a riskier option with a low expected utility might reveal urgency in achieving her goal. This might provide an opportunity to measure the decision maker’s wishes and also shed light on revealing the automatic and unconscious calculation in mind. It is suggested that an individual’s series of decisions be recorded in order to estimate their goal and urgency in achieving it. The goal in mind might be measured when the time question is answered, *e*.*g*., by the individual’s plan. The urgency might be obtained when the goal in mind is answered directly by the individual or estimated according to the individual’s need.

**Fig 8:**
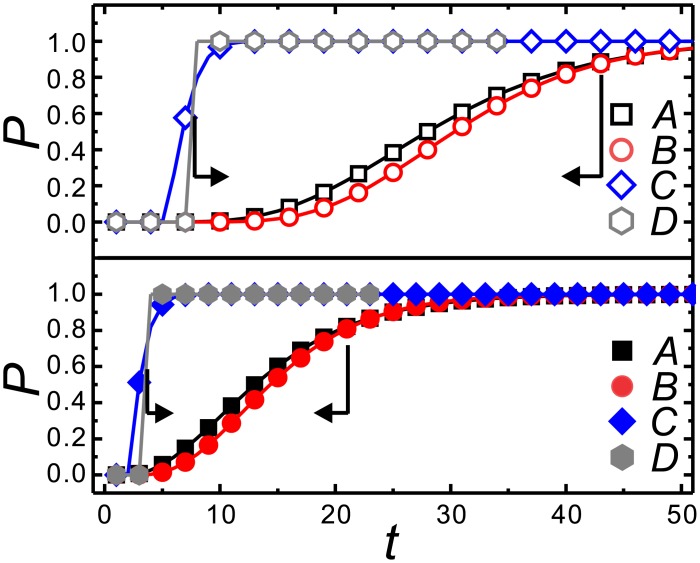
The CDF curves for decisions between *A*(4000, 0.2) and *B*(3000, 0.25), and between *C*(4000, 0.8) and *D*(3000, 1) with G of 24000 (upper panel) and 12000 (lower panel). A possible explanation resulted from our model − the majority of subjects that took *A* and *D* might imply that they wish to achieve a goal of 12000 within 5 < *t* < 20 or to achieve a goal of 24000 within 10 < *t* < 42. If these subjects wish to achieve the goal by *t* = 30, it could be estimated that the goal in mind is about 24000.

The aforementioned experiments conducted by Kahneman and Tversky [22, 31, 65, 66] are taken as an example to illustrate how to estimate the goal or urgency. Fig 8 shows the CDF curves for a decision between *A*(4000, 0.2) and *B*(3000, 0.25) and between *C*(4000, 0.8) and *D*(3000, 1) with **G** of 24000 (upper panel) and 12000 (lower panel). Accordingly, 65% of the subjects chose *A* between *A* and *B*, and 80% chose *D* between *C* and *D*. Our model provides a possible explanation for the majority of subjects who took *A* and *D*. We then try to find a range of *t* that satisfies both **P**_**comA**_ > **P**_**comB**_ and **P**_**comD**_ > **P**_**comC**_. If they wished to achieve the goal within 5 < *t* < 20 (10 < *t* < 42), a goal in mind could be estimated as 12000 (24000). On the other hand, if they stated that their goal were 12000 (24000), the *t* in which they wished to reach the goal could be estimated as 5 < *t* < 20 (10 < *t* < 42).

### Prospect theory revisited

By using our model, we try to reinterpret two aspects in prospect theory [22, 24–26, 31]: (1) the framing effect and (2) non-linear preference. The framing effect, as mentioned previously, describes that in frame of gains or losses, people tend towards risk aversion or risk seeking, respectively.

**Fig 9:**
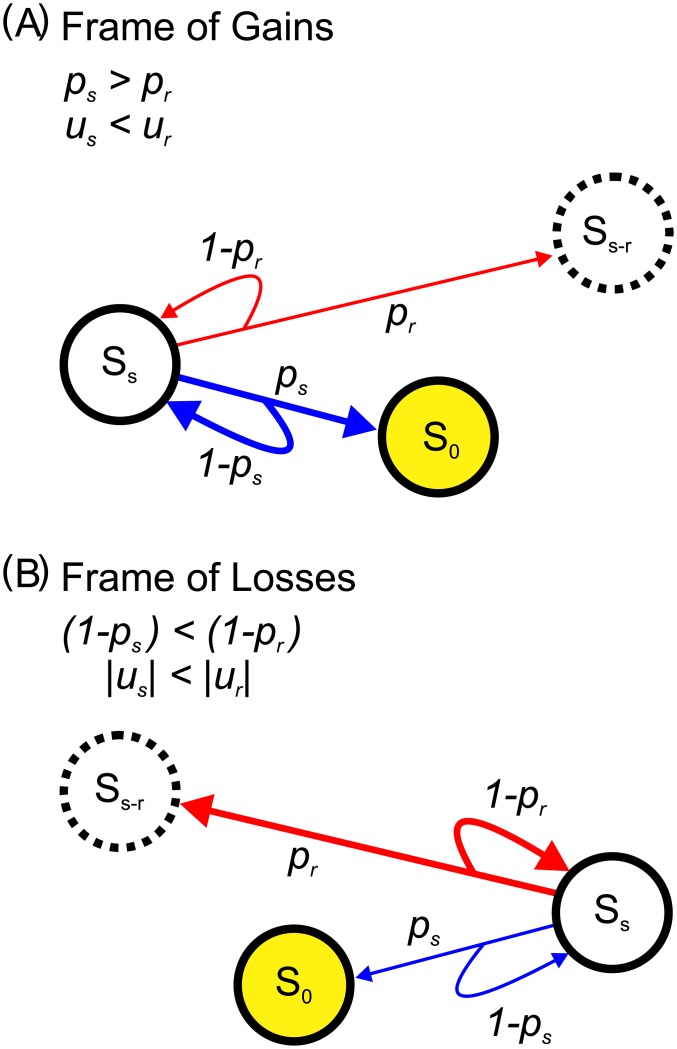
Examples of the mathematical interpretation of the economic behaviors in (A) frame of gains and (B) frame of losses. There are two options, a riskier *R*(*u*_*r*_, *p*_*r*_) and a safer *S*(*u*_*s*_, *p*_*s*_), in which *u*_*r*_ *p*_*r*_ = *u*_*s*_ *p*_*s*_ and *p*_*r*_ < *p*_*s*_. (A) In the frame of gains, both *u*_*r*_ and *p*_*s*_ are positive. When the decision maker is at S_*s*_, which is *s* stages away from the goal, S_0_, both options may lead her to S_0_. Nevertheless, choosing *S* allows her a higher probability to arrive S_0_ and a lower probability to stay at S_*s*_ than choosing *R*. Thus, *S* is preferable. This demonstrates a risk aversion behavior for a decision maker when the positive goal is near in the frame of gains. (B) In the frame of losses, both *u*_*r*_ and *u*_*s*_ are negative. When the decision maker is at S_*s*_, which is *s* stages away from S_0_ that she tries to avoid. Both options may lead her to S_0_. However, choosing *R* allows her to stay at S_0_ with a higher probability than choosing *S* ((1 − *p*_*r*_) > (1 − *p*_*s*_)). Although choosing *R* may lead her to S_*s*−*r*_, a worse stage than S_0_, it does not really matter since both S_*s*−*r*_ and S_0_ indicate her arrival to the negative goal. Additionally, choosing *R* may lead her farther away from S_0_. This demonstrates a risk seeking behavior for a decision maker when the goal is near in the frame of losses.

In a decision game with the payoff table as shown in Table 2. A decision maker that is *s* stages away from S_0_ has two options: a riskier option, *R*(*u*_*r*_, *p*_*r*_), and a safer option, *S*(*u*_*s*_, *p*_*s*_), in which *u*_*r*_ *p*_*r*_ = *u*_*s*_ *p*_*s*_ and *p*_*r*_ < *p*_*s*_. In the frame of gains as shown in Fig 9A, a decision maker is *s* stages away from S_0_. Choosing either *R* or *S* may lead her to S_0_. However, choosing *S* leads her to S_0_ with a higher chance than that choosing *R*. Besides, choosing *R* results in a probability of (1 − *p*_*r*_) to stay at S_*s*_, which is higher than (1 − *p*_*s*_) that choosing *S*. For a decision maker at the stage near the goal, it is preferable to choose a safer option. Therefore, one would exhibit risk aversion because there is no need to choose a riskier option with a small probability and a large reward that is in excess of the goal. In an extreme case, it is preferable to choose an option of a sure gain when the gain can just lead her to the goal with certainty.

**Table 2:** Reward and probability.

Option	*u*	*p*
R	*u*_*r*_	*p*_*r*_
	0	(1 − *p*_*r*_)
S	*u*_*s*_	*p*_*s*_
	0	(1 − *p*_*s*_)

We further consider the situation in frame of losses by assuming both u_*r*_ and u_*s*_ are negative. In Fig 9B, S_0_ is set as negative, indicates a lower bound of losses, *e*.*g*., the bankruptcy, which a decision maker tries to avoid. At S_*s*_, choosing *R* results in a chance of (1 − *p*_*r*_) to stay at S_*s*_, which is higher than (1 − *p*_*s*_) that choosing *S*. Therefore, the decision maker would show a preference in choosing a riskier option. That is, a decision maker may behave as a risk seeker in the frame of losses, especially when she is near the negative goal. Besides, a sure loss (*p*_*s*_ = 1) is not welcome since a rational decision maker tries to avoid the arrival at S_0_.

This demonstrates risk aversion in frame of gains and risk seeking in frame of losses. Such an asymmetry between the behaviors in the frame of gains and the frame of losses is embedded in our model. In other words, the framing effect in prospect theory can be mathematically interpreted.

The nonlinear preference, the concavity and convexity in the value-utility curve as shown in the prospect theory is referred to psychological activities [25]. In our model, the expected utility is in proportion to the monetary value. Hence, the criterion instead of the expected utility, **P**_**com**_(**t**) is intrinsically nonlinear, which may related to the nonlinear preference in the prospect theory.

### Mathematical interpretation of economical behaviors

The **house money effect** proposed by Thaler and Johnson [28] describes that people will tend to spend the money of a prior gain, *e*.*g*., earned from gambling, in a risk seeking mode. Our model can interpret that the one has a larger goal in dealing with this money. In other words, the one is willing to take a risk because the goal is out of the consideration. In the presence of prior losses, the decision maker will take the options with rewards that have opportunities to break even, which is called **break—money effect** [28]. This implies that with a prior loss, the goal might be shifted to *break even* or at least *get something* [65, 67, 68]. Such that, the tendencies toward a risk-seeking behavior would be enhanced especially in the frame of losses.

Besides, people may divide their money in parts, which will be used in different ways according to *mental accounts* [28]. Our model suggests that people can finely adjust the weights in a strategy for each account by the goal and the time to reach that goal. The computational shortcut is proposed to be included in heuristic procedure in decision making [8, 25, 69–72]. Our model suggests that people might unconsciously proceed such a mental computation on the **P**_**com**_.

### Perspectives

An individual’s behavior or activity may play a crucial role in the collective cooperation. It is proven that the social diversity of individual behavior will substantially improve the level of cooperation [73–76]. Presently, our model can be considered as a single player game, which provides a reference for the behaviors of individuals in decision making. Such that, the game theory can involve by considering the players’ goals and urgencies based on our model. Additionally, the policy maker may design a game situation to enhance the level of cooperation in order to obtain the public good with less contradiction.

For making decisions under uncertainty, where the (*u*, *p*) for each option is unknown. Learning from experience is thus important in constructing the (*u*, *p*) practically and in explaining the human behaviors [55, 77]. Our model provide a mathematical process to estimate the (*u*_*i*_, *p*_*i*_) for all possible options. As shown in Eq 6, **A** can be resolved by the resulting **P**_**com**_(**t**) by setting a trial **W** after repeated decision-makings. This provides an opportunity to find the decision network, possible combinations of the (*u*_*i*_, *p*_*i*_)s, and the associated **Q**s through these trials. Therefore, learning from experiences becomes mathematically achievable.

The proposed model combines “the goal and time” together to provide a mathematical procedure in decision making and also provides a mathematical foundation to explain several psychological effects in economical behaviors. Additionally, such a mathematical procedure also provides foundation for decision making by Artificial Intelligence.

## Conclusion

The proposed network approach provides a powerful tool for analysing the time-dependent outcomes of decisions. The behaviors that contradict those predicted by expected utility theory can be mathematically explained. Our results imply that planning could be embedded in decision behaviors. For a rational decision under risk, one needs to consider achievement of the desired goal within a specific period in the future rather than maximizing the expected utility in the present. Moreover, the model not only provides a mathematical foundation for resolving ambiguity in decision making, but also provides mathematical reasoning on the behaviors that chooses the options with lower expected utilities.
